# Microstructural Characteristics of Cement-Based Materials Fabricated Using Multi-Mode Fiber Laser

**DOI:** 10.3390/ma13030546

**Published:** 2020-01-23

**Authors:** Youngjin Seo, Dongkyoung Lee, Sukhoon Pyo

**Affiliations:** 1 Department of Mechanical and Automotive Engineering, Kongju National University, Cheonan 31080, Korea; syjvlfry1004@gmail.com; 2 School of Urban and Environmental Engineering, Ulsan National Institute of Science and Technology (UNIST), Ulsan 44919, Korea

**Keywords:** cement-based materials, laser cutting, multi-mode fiber laser, microstructure, SEM/EDX analysis

## Abstract

Cement-based materials are the most prevalent construction materials, and the conventional cutting techniques are still mostly used for fabricating the materials. However, these conventional cutting methods could generate undesirable micro-cracks and remove unintentional structural sections. This experimental study aims to evaluate the effects of the new fabricating method using laser on the microstructural characteristics of the cement-based materials. The experimental variables are laser cutting speed, water to cement ratio and material compositions. In order to compare the microstructure before and after the laser interaction, the microstructure of the cut surface is observed through scanning electron microscopy/energy dispersive X-Ray (SEM/EDX). After the laser interaction, the Material Removed Zone (MRZ) and Heat Affected Zone (HAZ) are observed on the cut surface. In MRZ, it is found that the glassy layer is thickened by an increasing amount of silicate-based materials in cement-based materials. In addition, it concluded that the amount of silicate-based material mixed in the cement-based materials affects the laser cutting quality.

## 1. Introduction

Cement-based materials are prevalent materials in almost all construction fields, such as buildings, roads, bridges, dams and nuclear power plants. However, cement-based materials can be deteriorated under some unfavorable circumstances, such as high chloride environments, poorly controlled concrete casting and unexpected overloading. For this reason, the demand for the removal of deteriorated parts is steadily increasing. Currently, conventional cutting methods, such as drill cutting, water jet and diamond saw cutting, are still in use [1,2,3,4,5]. Conventional cutting methods are time consuming and relatively low precision. It shows uncertain removal about deteriorated concrete. In addition, undesirable vibration could cause micro-cracks in the sound concrete structure, which could lead to the removal of essential concrete sections. Furthermore, there are additional drawbacks, including noise and dust generation during the concrete removing process.

Laser-Aided Manufacturing (LAM), on the other hand, has many advantages, and is therefore used in a variety of applications such as automotive, aerospace, electronics and semiconductors [6,7,8,9,10,11,12,13,14,15]. LAM has an exceptionally high energy density and can be focused to highly localized areas. Furthermore, there is no physical contact between the laser head and the work piece. An accurately focused laser beam allows a low Heat-Affected Zone (HAZ) and precise manufacturing. Especially since the heat-affected portion generated by the laser is very small, the possibility of material deterioration is low. Therefore, LAM is very efficient and yields precisely controlled products. Furthermore, the laser provides a high energy density and can manipulate the optics to focus on very small area. As a result, lasers can be applied to a wide range of materials such as wood, composites, rubbers and metals.

By applying these advantages, Lee et al. [16,17] applied a laser cutting technique to cement composites using a 1-kW multi-mode fiber laser (IPG YLS-1000). In addition to the geometrical parameters, the Energy Dispersive X-Ray (EDX) component analysis was used to study the compositional changes before and after the laser cutting of the composite surface. Based on these relationships, they found the laser parameters, including the laser scanning speed and the line energy required to completely cut 4 mm-thick cement paste. Moreover, the components and properties of cement mortar surface after laser cutting were analyzed. It was also pointed out that the addition of silica sand, silica fume and silica powder to the basic cement composite results in poor quality during the laser cutting.

Crouse et al. [18] cut a 150 × 150 × 45 mm concrete slab using a 1.2 kW CO_2_ laser (Rofin-Sinar RS-1000) and a 1.5 kW high-power diode laser (LDL-160-1500). The CO_2_ laser and diode laser were irradiated repeatedly to observe the cutting width and the number of passes when the penetration depth was 120 mm. Numerical simulation was also applied to investigate the effect of beam characteristics on kerf convergence during multi-pass processing. They concluded that the high-power diode laser is more suitable than a CO_2_ laser for deep-section concrete cutting. Because the rectangular beam shape of the diode laser is wider and more parallel, its cutting width remains relatively constant while the penetration depth is increased.

Muto et al. [19] used optic beam delivery and fiber laser (IPG, YLR-5000) to cut the concrete. They used the ytterbium multi-mode laser of 1070 nm wavelength with a 1 km fiber-optic cable, and set a focal point at 0.75 mm from the surface of the specimen through a 120 mm diameter lens. The thickness of the concrete slab was 100 mm and the scanning speed was 5 mm/s. In this study, the applicability of laser-based concrete cutting technology is suggested by specifying the diameter of the lens and the length of the fiber optic cable. Most of the previous researches have studied the laser parameters and compositional changes of concrete cutting to cut concrete. However, the change in the microstructure of concrete before and after laser cutting has been not thoroughly studied. Furthermore, after the laser cutting, the investigation on microstructures of the fabricated surface of the cement-based materials has not been proceeded.

Therefore, this study investigates the effect of the various material compositions on the microstructure of cement-based material after laser cutting. Laser cutting of cement-based materials is carried out using a continuous wave (CW) multimode fiber laser. The cutting characteristics and microstructure of the cut surface of the laser-treated region are examined by Scanning Electron Microscope (SEM). EDX is performed to observe the chemical composition changes in the cut region. In addition, the Heat Affected Zone (HAZ) of the microstructure is analyzed by dividing the area where the material is removed from the cut surface and the area where the material is not removed, and the distribution of the specific ingredient is discussed.

## 2. Research Significance

This study, for the first time, reports the interaction between lasers and cement-based materials through the microstructural characterization of a cut surface after the laser cutting. It is significant because the relationship between laser and cement-based materials is essential to be fully utilized and applied in laser-aided manufacturing, such as laser cutting, drilling and scabbling.

Also, this study would be able to help us to understand the microstructural characteristics of cement-based materials when laser processing is performed, which would contribute to various fields related to the cement-based materials and laser processing.

## 3. Materials and Mix Design

In order to investigate the effects of the mixture design on concrete, many studies have been conducted. Ostrowski et al. [20,21] evaluated the effect of aggregate shape on the mechanical properties of self-compacting high-performance fiber-reinforced concrete (SCHPFRC). They characterized the difference between the regular coarse aggregate and irregular coarse aggregate by comparing the rheological, physical and mechanical properties. Pyo et al. [22] proposed the effect of the coarser fine aggregate, including dolomite and basalt on ultra-high-performance concrete (UHPC) based on the particle packing theory. They obviously indicated the mechanical properties and shrinkage of UHPC through mechanical test and dimensional stability.

The material used in this study is Ordinary Portland Cement (OPC), silica sand with about 93% SiO_2_ weight, silica powder, undensified silica fume (Elkem 940U) and polycarboxylate-based superplasticizer. Silica sand Ⅰ and Ⅱ with median size of 0.15 mm and 0.53 mm were used, respectively, as fine aggregates in cement-based materials. Silica fume and silica powder (median size of 3.15 μm) are used to increase the strength of cement-based materials through pozzolanic reaction and additional particle packing, respectively.

Three types of cement-based materials with the specimen thickness of 50 mm were prepared, and the specimens were named as follows. Laser-processed Paste (LP) is cement paste mixed with water and cement, Laser-processed Mortar (LM) is cement mortar mixed with water, cement and silica sand. Laser-processed UHPC (LU) is UHPC mixture made by mixing silicate-based materials such as silica sand, silica powder and silica fume [23]. LP and LM were selected because cement paste and cement mortar were the basic cement-based material units. LU was selected to compare the effect of laser on the additional silicate materials, silica fume and silica powder. In addition, LP and LM were set as a water–cement ratio of 0.25, 0.35 and 0.4. LU set the different weight of silica fume and silica powder, respectively. The detailed mixture design and compressive strength can be found in Lee et al. [16].

## 4. Experimental Setup

The specimens used in this study were mixed using a laboratory concrete mixer. After mixing the cement and water, the mixture was poured into 50-mm cube molds to produce a specimen. After casting, the prepared specimens were covered with a plastic sheet, stored at room temperature for 24 h. The demolded specimen was submerged in a water tank for 28 days of curing. The water cured specimens were dried and we conducted the laser cutting test.

**Figure 1:**
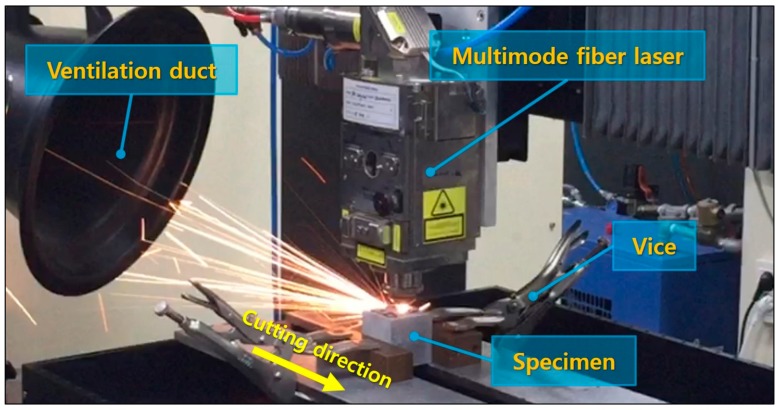
Experiment set-up.

The experimental setup is shown in Figure 1. The multi-mode continuous-fiber laser (IPG-YLS-10000 MM) has a laser beam diameter of 150 μm, and the laser focus is adjusted to the surface of the concrete specimen. The laser wavelength is 1070 nm, the maximum available output is 10 kW, and the N_2_ assistant gas is fixed at a pressure of 7 bar. The specimens are placed on the test bed and fixed with a vice; whereas, this table-mounted system might not be required in actual applications due to relatively large-scaled fixed concrete structures. The test bed is composed of two metal plates and is arranged horizontally with the interval of 6.5 mm to remove the molten cement particles. The laser beam is irradiated perpendicular to the cement composite from the moving head. A ventilation duct is placed on the opposite side of the laser moving direction to collect dust particles that occurred during experiments. The laser power is set to 9 kW, and the laser scanning speed is designated as the only controllable parameter to simplify the experimental parameters. The laser cutting speed is reduced from 4 m/min to 1 m/min at 1 m/min intervals. Additionally, the laser cutting speed of 0.5 m/min and 0.25 m/min are used.

## 5. Result and Discussion

### 5.1. Microstructure of the No-Processed Cement-Based Materials

**Figure 2:**
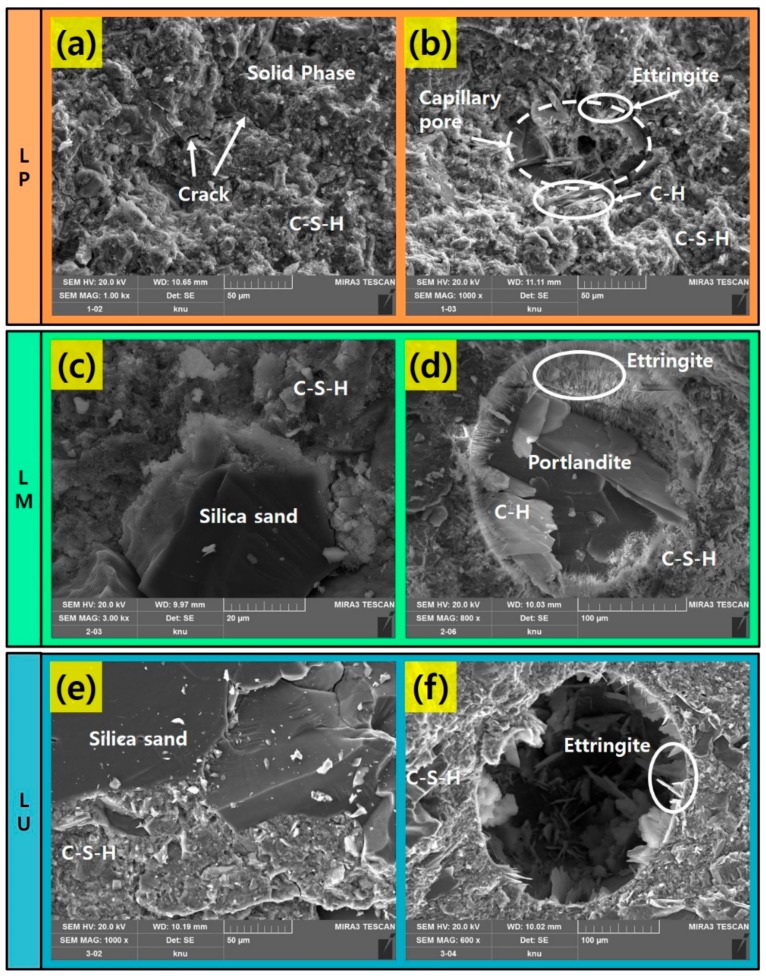
SEM images of the specimen before the laser cutting: (**a**) LP; (**b**) pore on LP; (**c**) LM; (**d**) pore on LM; (**e**) LU and (**f**) pore on LU.

SEM is used to study the influence of the amount of silicate-based materials on the microstructure of the cement-based materials. Figure 2 shows SEM images of the cement-based materials to observe the microstructure of the materials before the laser irradiation. The heterogeneous distribution of Calcium Silicate Hydrate (C–S–H) and Calcium Hydroxide (C–H) and needle-shaped ettringite are observed in the fracture surface of the cement-based materials. In addition, micro cracks are visible in surface. The SEM images of LP (Figure 2a), LM and LU (Figure 2c,e) display the hardened cement pastes. At the same time, some silica sands are observed in LM and LU. Deposits of hydrated cement products are dispersed around the pores (Figure 2b,d). On the other hand, relatively low amounts of C–H and ettringite are found in pores of the LU compared to other specimens (Figure 2f). This is because the LU is made by mixing silica fume, which accelerates the cement hydration reaction rate and causes C–S–H gel to increase rapidly [24,25,26].

### 5.2. Microstructure of the Processed Cement-Based Materials

The microstructures of laser-irradiated, cement-based materials are analyzed by SEM/EDX. To facilitate the SEM/EDX, the laser-irradiated specimens are fabricated with the size of 10×27×10 mm. 

**Figure 3:**
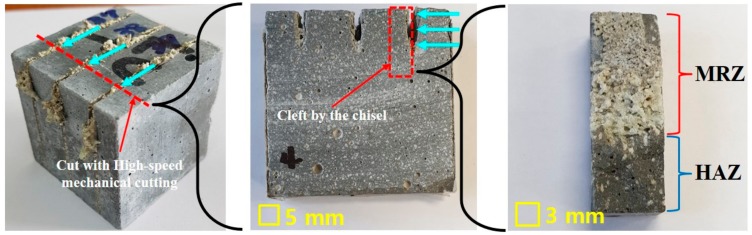
Fabrication process of a laser-processed specimen used scanning electron microscopy/energy dispersive X-Ray (SEM/EDX).

Figure 3 shows a fabrication process of the specimen after laser irradiation. First of all, the cement-based material is cut by the high-speed mechanical cutting. After the cutting, the specimen is split by the chisel to observe the cut surface affected by laser. If a mechanical cutting machine is used during this process, the microstructure of the cut surface on the specimen is affected by the heat generated during the mechanical cutting. Therefore, the specimens are cleft by the chisel and used for SEM/EDX analysis. In addition, the cut surface of specimen is classified into the Material Removed Zone (MRZ) and Heat Affected Zone (HAZ). MRZ is the area where the material is vaporized and removed by laser. On the contrary, HAZ is the area where material is not removed and only affected by heat [16,17]. Since the perfectly cut specimens exist only in the MRZ at the cut surface, the partially penetrated specimen is used to compare MRZ with HAZ.

**Figure 4:**
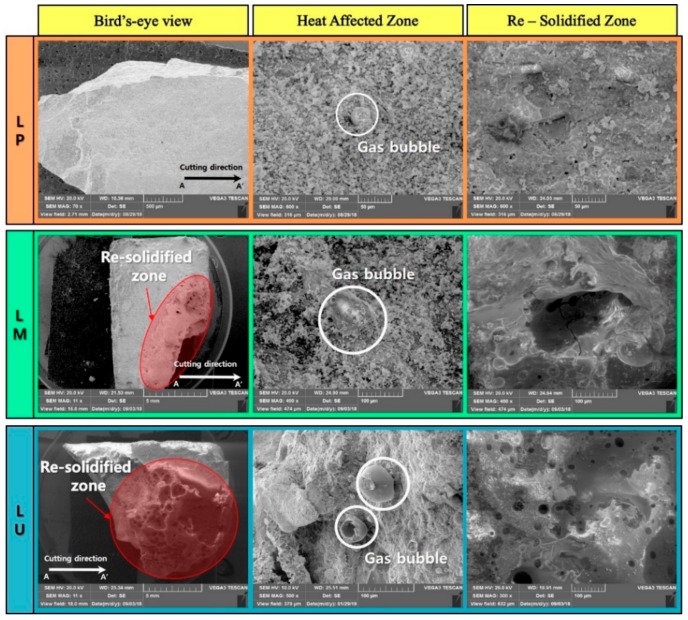
SEM images of the cut surface on the laser-processed specimen after the laser cutting.

**Figure 5:**
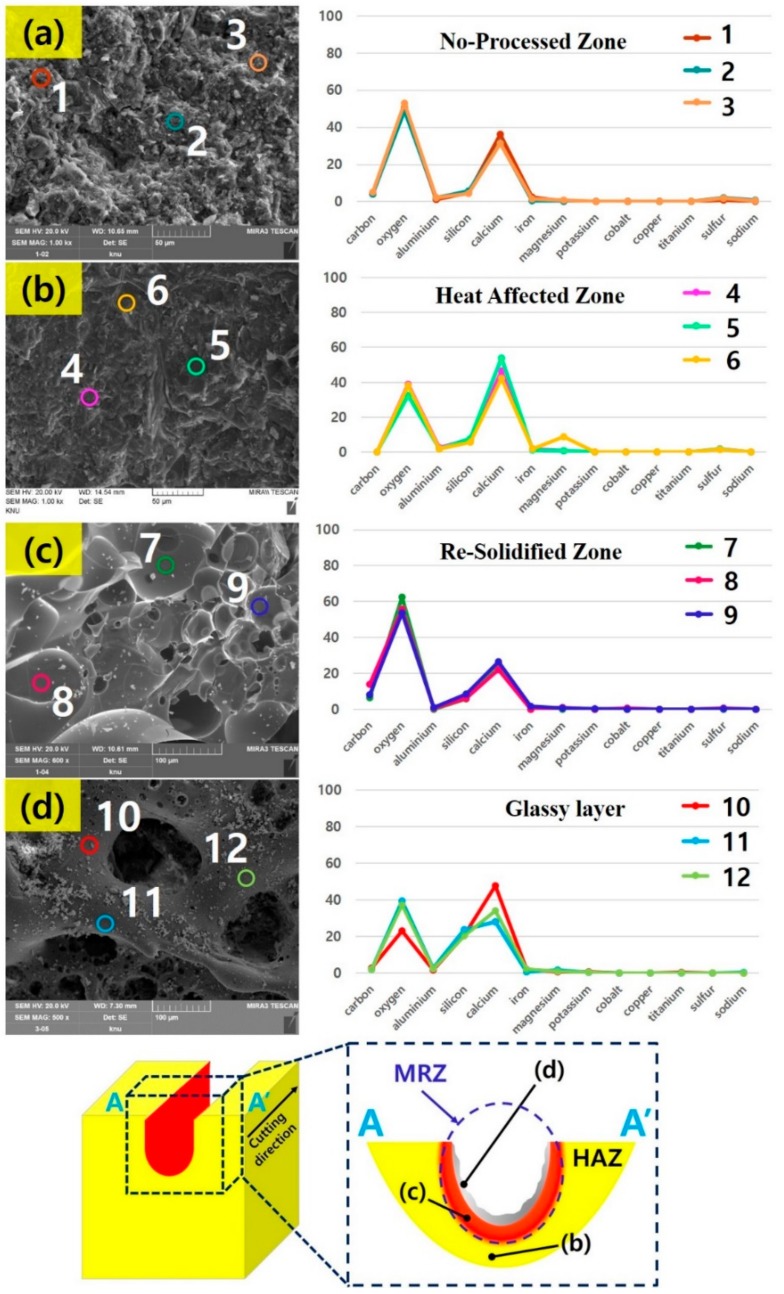
Energy dispersive X-Ray (EDX) analysis of (**a**) No Processed zone; (**b**) Heat Affected zone; (**c**) Re-solidified zone; (**d**) glassy layer in LU.

To observe how the laser affects the microstructure of the cement-based material, the SEM images of the cut surface on the laser-processed, cement-based material is shown in Figure 4. HAZ can be seen in most specimens, and gas bubbles are observed in this region. The gas bubble can be formed by the heat generated by the laser when the cement hydration products are dehydrated, e.g., the calcium carbonate is generated and is released into the air by the decomposition of calcium hydroxide under the heat [27]. Unlike LM and LU, LP shows the least Re-Solidified Zone, which is the resolidified area after the specimen is melted by the laser. Additionally, small pores of less than 10 μm are observed compared to other specimens. In the LM, the resolidified zone is partially observed, and pores of about 30 μm or more are noted in the glassy layer. The glassy layer is the layer structure in which the silicon with a high vaporization point is in part evaporated by the laser and is resolidified from the molten state. The LP formed a small amount of silicon, so the glassy layer was not formed. Unlike LP, both LM and LU were mixed with silicate-based materials such as silica sand, silica fume and silica powder. As the amount of silicate-based materials increases, the thickness of the glassy layer increases. To observe the change of the chemical composition in the laser irradiated area, the components of the no-processed zone, glassy layer, HAZ and re-solidified zone are analyzed through EDX, and it is shown in Figure 5.

To investigate the effect of the laser on the chemical change of the cut surface on cement-based material, EDX component analysis of the cement-based materials is performed after classifying four regions, e.g., no-processed zone, HAZ, re-solidified zone, glassy layer. In addition, the LU is selected for EDX analysis because this specimen provides those four regions clearly. Before the laser irradiation, the no-processed zone of the LU is mostly composed of oxygen (50.84%) and calcium (33.09%), as shown in Figure 5a. After laser irradiation, the weight percent of the components of the LU specimen varies depending on the microstructure configuration. In HAZ, the oxygen weight percentage decreased from 50.84% to 36.58%, but the weight percent of calcium increases from 33.09% to 47.55% as shown in Figure 5b. This is the result of the evaporation of water by the heat generated by the laser.

In the Re-solidified zone, 56.62% of oxygen is observed, which is the maximum weight percent compared to the other regions, but the calcium is observed with the minimum weight of 23.45%, as shown in Figure 5c. It is expected that the emission gas generated by the laser is trapped as the molten concrete is re-solidified. The glassy layer is composed of more than 20% silicon, 37.47% oxygen and 29.55% calcium (Figure 5d). In addition, calcium is vaporized at the low vaporization point of 1484 °C. However, the silicon has not been vaporized in the molten cement-based materials due to the high vaporization point of 3265 °C. Therefore, the weight percent of silicon is high in the glassy layer compared to other regions.

### 5.3. EDX Mapping Analysis on Ingredient Distribution

**Figure 6:**
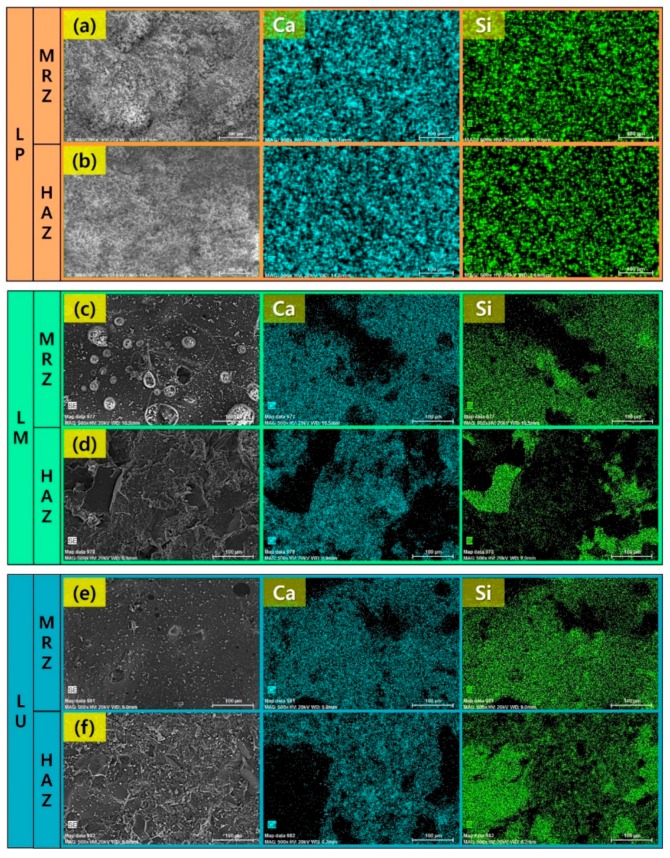
Elemental mapping for Ca and Al on the specimens after the laser cutting: (**a**) MRZ of LP; (**b**) HAZ of LP; (**c**) MRZ of LM; (**d**) HAZ of LM; (**e**) MRZ of LU; and (**f**) HAZ of LU.

EDX mapping analysis of cement-based materials is shown in Figure 6. MRZ are directly affected by the lasers, and the material is removed as shown in Figure 6a,c,e. On the other hand, HAZ is not removed and is only affected by heat conduction (Figure 6b,d,f). In case of the LP specimen, the compositional distribution of calcium and silicon is similar to the microstructures of the MRZ and HAZ. On the other hand, in the LM and LU specimen, the components of calcium and silicon are similar in MRZ. However, in HAZ, silicon is observed in areas without calcium. The cement and silicate-based materials are melted due to the laser irradiation, and then mixed with each other. After that, the molten concrete is resolidified in a mixed state. Therefore, calcium and silicon show a similar distribution in the MRZ. On the other hand, calcium and silicon in the HAZ do not mix with each other since the temperature of the cement-based materials remained in a solid phase. Thus, the LM and LU are mixed with silicate-based materials so that the distribution of calcium and silicon is clearly observed. However, in the LP specimen, this distribution is not apparent due to the cement paste being mixed only with cement and water.

## 6. Conclusions

This study is performed to observe the effect of laser cutting on the microstructure of cement-based materials. Three types of cement-based materials are fabricated by selectively mixing silica sand, silica powder and silica fume. The laser power set as the 9 kW. The physical and chemical changes in the cut surface on cement-based materials before and after laser irradiation are observed. Furthermore, the reaction characteristics between laser and cement-based materials are evaluated by investigating microstructure changes through the SEM/EDX analysis.

1. In the microstructure of cement-based materials, cement hydrates such as C–S–H, C–H, and ettringite generated by the cement hydration reaction were observed in the cement-based materials before laser irradiation.
2. After the laser cutting, three characteristics are observed, such as the re-solidified zone, HAZ and the glassy layer in the cut surface of cement-based materials.
3. The glassy layer is not observed in cement paste among three types of specimens. In addition, the comparison of the results of cement mortar and UHPC show that the glassy layer is thicker as the silicon weight percent increases.
4. Under the tested laser parameters, the amount of silicate-based material mixed in the cement-based materials affects the laser cutting quality.

It should be noted that a series of studies on the effect of the laser on cement-based materials, such as normal performance and high performance concrete, need to be proceed as the future research of this study for clarifying the laser mechanisms on cement-based materials through changes of mechanical properties and chemical compositions under laser interaction. Furthermore, additional research should be carried out in the future research to identify the effect of cutting speed on the microstructural characteristics of cement-based materials fabricated using a laser.
